# Hyalinizing Clear Cell Carcinoma of the Base of the Tongue: A Case Report and Literature Review

**DOI:** 10.7759/cureus.81249

**Published:** 2025-03-26

**Authors:** Shuichi Matsumoto, Taihei Kajiyama, Hiroaki Ito, Hironaga Satake, Tomoki Kimura, Masanori Teshima

**Affiliations:** 1 Otolaryngology - Head and Neck Surgery, Kochi Medical School Hospital, Kochi University, Nankoku, JPN; 2 Medical Oncology, Kochi Medical School Hospital, Kochi University, Nankoku, JPN; 3 Radiation Oncology, Kochi University, Kochi, JPN

**Keywords:** base of tongue cancer, clear cell carcinoma, salivary gland carcinoma, salivary gland tumor, trans oral video-assisted surgery

## Abstract

Hyalinizing clear cell carcinoma (HCCC) is a rare low-grade malignant tumor composed of clear cytoplasmic cells. It mainly arises from the minor salivary glands of the oral cavity and oropharynx of the head and neck region. As a relatively slow-growing tumor, the main symptom of HCCC is indolent discomfort associated with enlargement and little local pain. This report presents a 33-year-old female patient with HCCC at the base of the tongue and reviews 12 cases of HCCC occurring at the base of the tongue with a surgical procedure in detail. In most cases, excision was performed using the mandibulotomy or a transcervical approach. However, it was a small, low-grade malignant tumor in this case, and the transoral approach was adopted. This minimally invasive procedure allows early oral intake and a short hospitalization period. The oral method is also considered a useful surgical approach for treating HCCC of the base of the tongue.

## Introduction

Hyalinizing clear cell carcinoma (HCCC) is a carcinoma composed of clear and eosinophilic cells within variably hyalinized stroma that mainly originates from oral minor salivary glands in locations that include the hard and soft palate, accounting for 1% of all salivary gland cancers as described in 1994 [1-3]. HCCC is a slow-growing, low-grade malignant tumor with a good prognosis. [3]. In this report, we describe a rare case of HCCC at the base of the tongue, with evidence of regional lymph node (LN) metastasis, for which transoral surgery was performed. This report includes a literature review of similar cases to highlight the diagnostic and treatment management challenges.

## Case presentation

**Figure 1:**
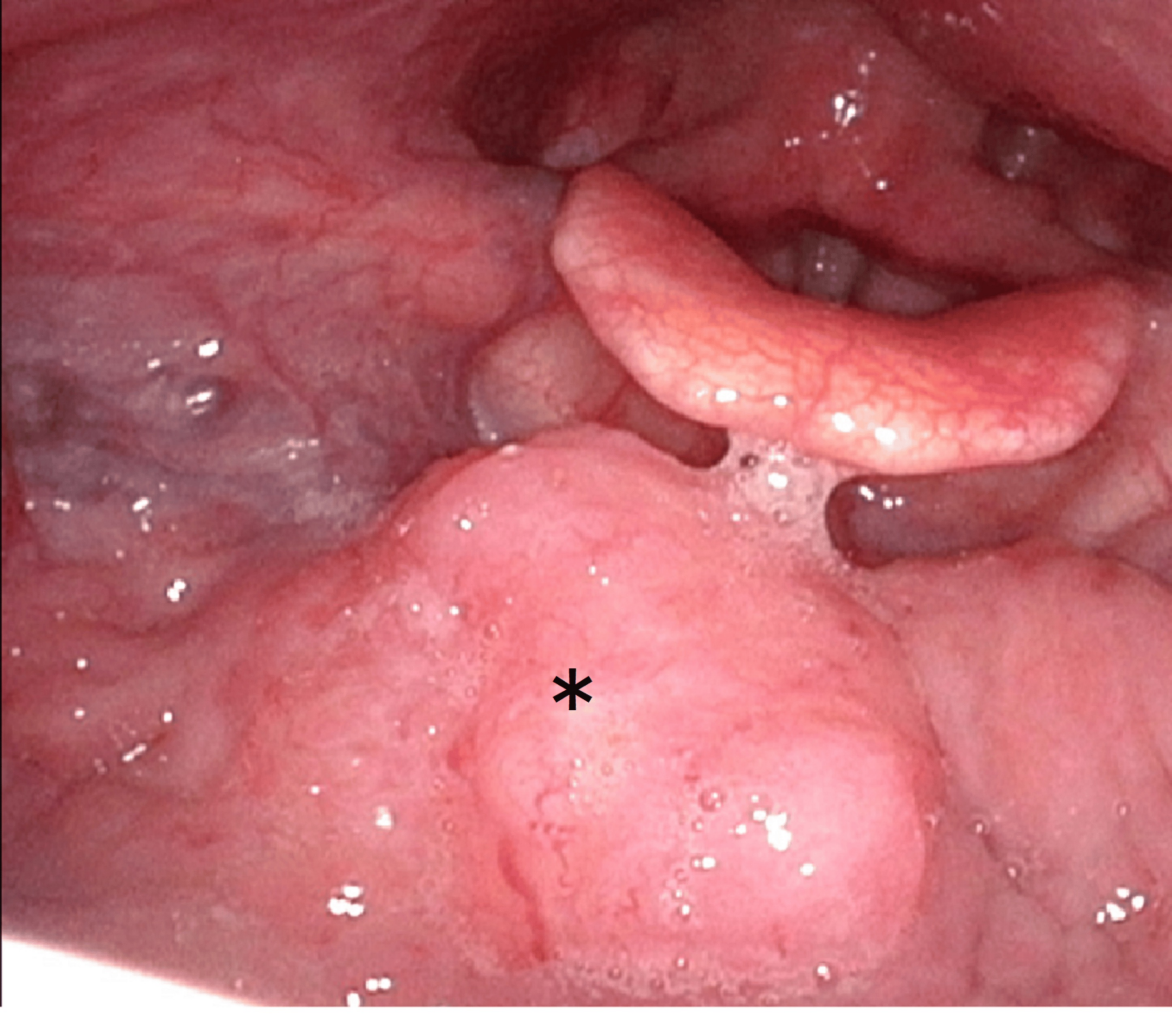
Local view of the mass in the base of the tongue ＊Star indicates clear cell carcinoma of the base of tongue

A 33-year-old female patient presented with right submandibular swelling over 2-3 months with a gradual tendency to increase. She had a slight fever for several days and visited her local physician's office. She had multiple right upper deep internal cervical LN swelling and slight midline swelling at the base of the tongue without any other abnormality in the head and neck region (Figure 1). CT showed LN swelling in the right upper neck, but no other abnormalities were seen, including at the base of the tongue. An incisional biopsy was performed to diagnose the cervical LN swelling, and the pathological result indicated HCCC. Next, another biopsy was performed on the enlarged base of the tongue, and the same diagnosis of HCCC as the cervical LN was obtained.

Furthermore, genetic analysis of the cervical LN tissue was positive for Ewing Sarcoma Breakpoint Region 1 (EWSR1) gene fusion. As a possible primary site, the HCCC was thought to be from the minor salivary glands at the base of the tongue and LN metastasis on the right neck because HCCC from major salivary glands accounts for only a small percentage. However, the base of the tongue is the second most common site of HCCC after the soft palate.

**Figure 2:**
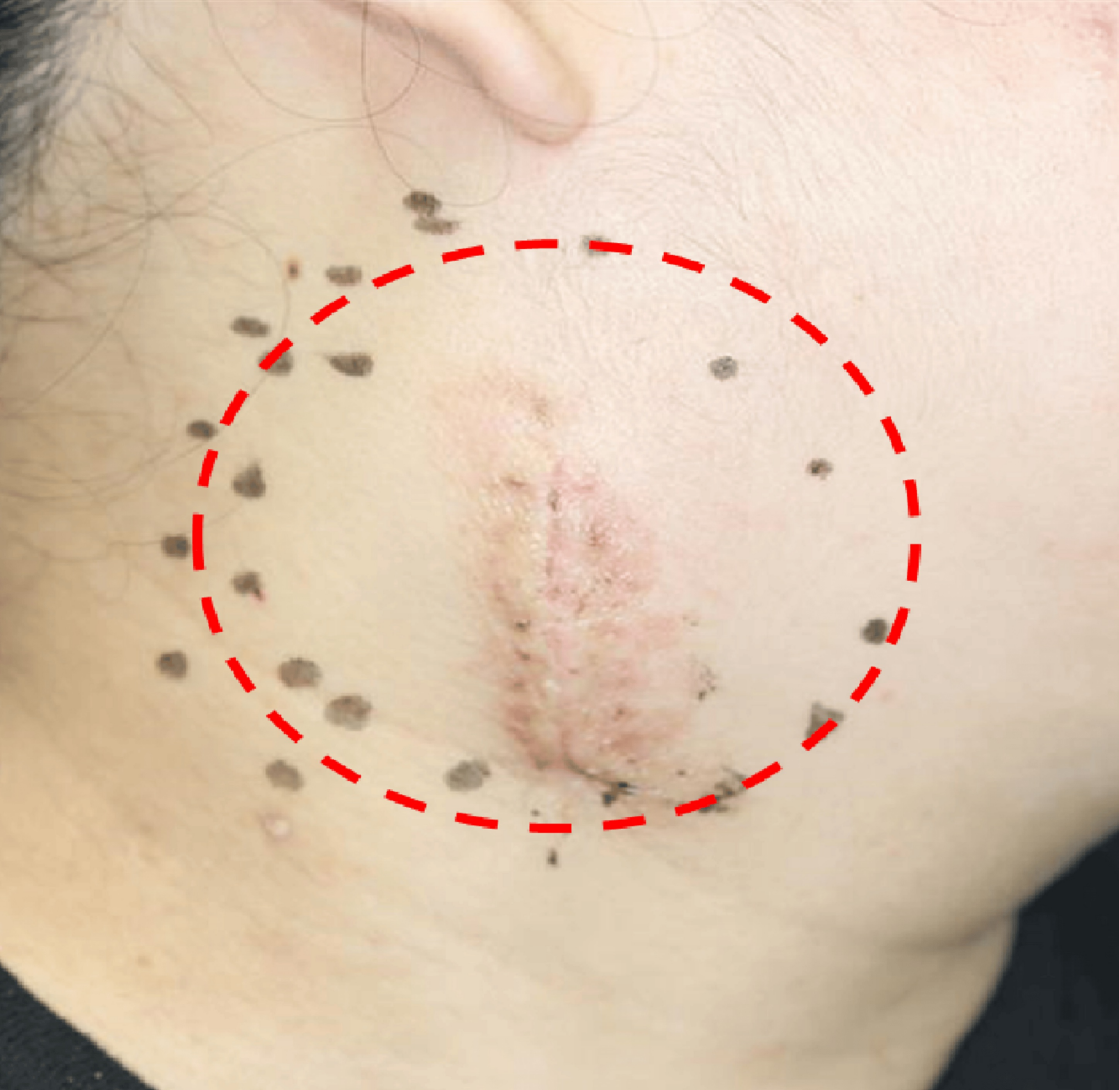
Right cervical lymph node incision biopsy scar. The red dotted line shows the extent of cervical lymph node enlargement.

The patient was then referred to the previous department of otolaryngology for consultation. At the initial visit, a 7 cm swelling was seen in the right submandibular region, and an external incisional biopsy wound was found in the center of the swelling (Figure 2).

**Figure 3:**
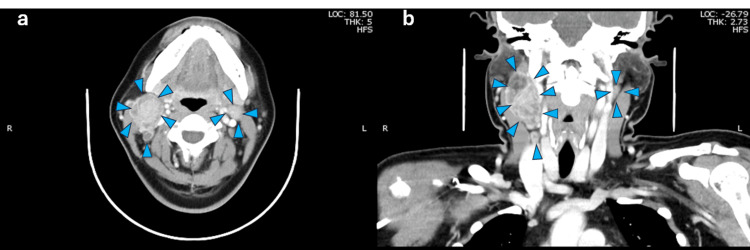
Contrast-enhanced computed tomography in case Figure 3a: Axial view, Figure 3b: Coronal view Bilateral lymph node metastasis are surrounded by blue arrowheads

**Figure 4:**
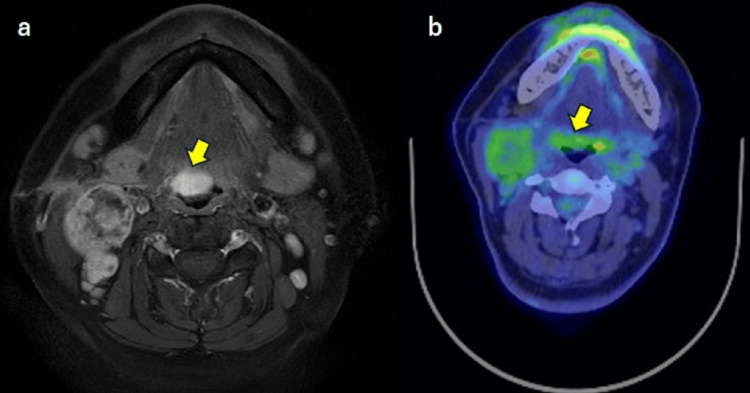
MRI axial view T1 with Gd enhancement and PET/CT axial view The arrow indicates a tumor on the base of the tongue and metastasis in bilateral cervical lymph nodes. PET/CT: Positron emissions tomography/computed tomography; Gd: Gadolinium

CT and MRI revealed a tumor extending from the median to the right side of the base of the tongue. (Figure 3-4), with a smooth surface and without ulcers and bleeding. The tumor size at the base of the tongue was 17 x 10 mm, with a deep invasion of about 15 mm. A positron emission tomography (PET) scan showed pale positron emission in the primary tumor and the cervical LN (Figure 4). Based on the above, we definitively diagnosed oropharyngeal carcinoma of the anterior wall, HCCC, T1N2cM0, and Stage IVA.

The patient was scheduled for surgery as a curative treatment. The surgical approach involved oral excision of the primary tumor because the biopsy tissue had already confirmed the diagnosis of HCCC as a low-grade cancer with a relatively small tumor size and little tendency to invade deep into the base of the tongue. In the case of a difficult oral resection, we prepared for transcervical external incision and mandibulotomy. After inducing general anesthesia by nasal intubation, a tracheotomy was performed, and the intubation tube was replaced. For the primary lesion, the oral cavity was expanded with the FK-WO retractor (laryngopharyngeal retractor), the base of the tongue was visualized using the endoscope, and the lesion was excised using a long energy device. We planned a safety margin of 5 mm from the tumor at the base of the tongue, and deep resection was performed at the depth of the hyoid bone. Operating time was 8 hours 37 minutes, and blood loss was 90ml. For cervical LN metastases, bilateral II-IV neck dissection was performed. The sternocleidomastoid muscle-infiltrated area on the right side was resected with partial complications.

**Figure 5:**
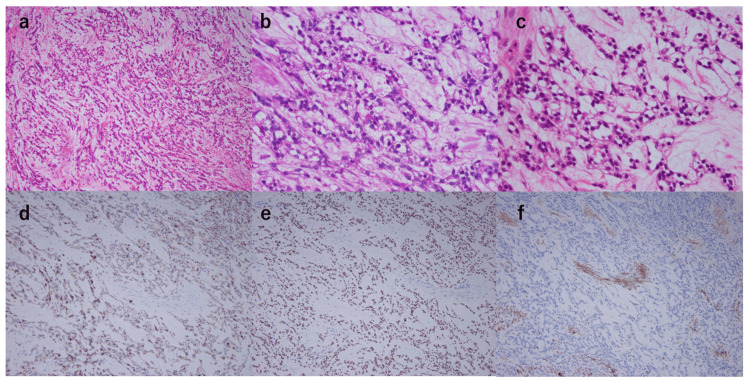
Hematoxylin and eosin (H&E) stained sections and immunohistochemical stained sections a: HE×100: Tumor cells arranged in a cordate pattern with a wide stroma. Some vitalization was also observed b: HE×400: Pale cells with bright cytoplasm are seen c: PAS: PAS-positive glycogen granules in the cytoplasm of bright cells. d: CK 5/6: The tumor cells were positive for CK 5/6 e: p63: The nucleus of tumor cells was positive for p63 protein f: aSMA: The stroma of tumor cells was negatively stained in places

**Figure 6:**
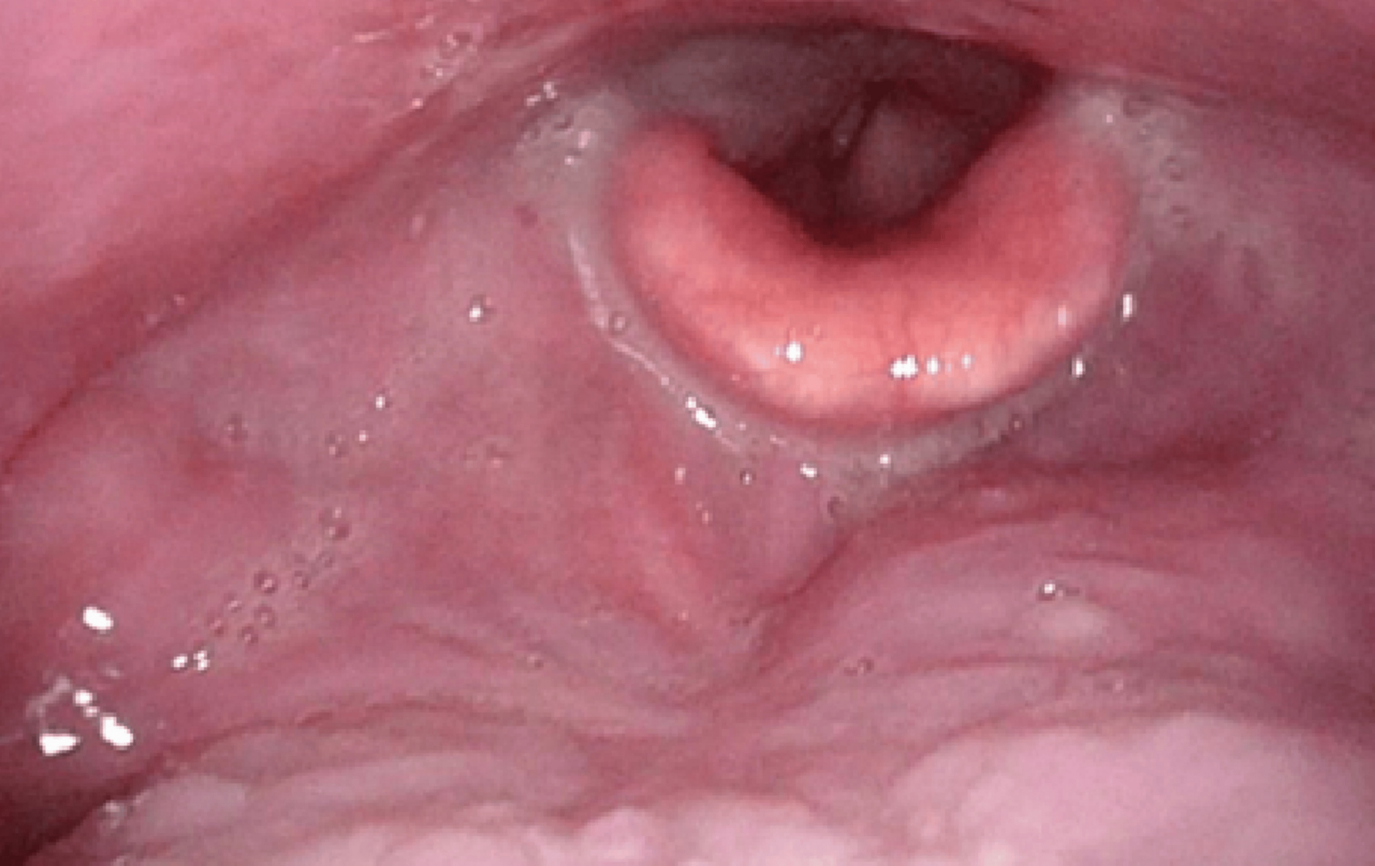
Findings at the base of the tongue after treatment No evidence of residual tumor at the base of the tongue

Postoperatively, the patient was managed with nasogastric tube feeding, started drinking water on day six, and could eat on day seven. The tracheostomy was changed to a simple tracheal cannula for home use on postoperative day six, and the patient was discharged home on postoperative day eight. Pathological examination revealed a primary tumor 25×17×15 mm in size, with a cord-like proliferation of pallid cells and a positive periodic acid- Schiff (PAS)-stain mucus in the cytoplasm (Figure 5). Immunostaining was positive for the epithelial markers CAM 5.2, CK5/6, CK14, and CK19, as well as p40 and p63. In contrast, the myoepithelial markers, including epithelial membrane antigen (EMA), alpha-smooth muscle actin (αSMA), and calponin, were all negative. Since the patient had an oropharyngeal carcinoma, p16 staining was also performed, but it was negative. The lymph node metastasis on the right Level IIA was 65 mm in size with microscopic extranodal invasion. A total of five metastatic lymph nodes were only on the right side. Pathological staging was pT2N3b. The previous physician confirmed that the patient had HCCC, including a positive EWSR1 gene fusion. The patient had lymph node metastases with extranodal invasion and a positive margin of the base of the tongue tumor, so we decided on postoperative chemoradiotherapy. Postoperative intensity-modulated radiotherapy was performed with standard radiation of 70 Gy (2 Gy/fraction, once a day, 5 times a week). Concomitant chemotherapy was given with cisplatin (CDDP; 40 mg/m2) weekly, based on the postoperative treatment of squamous cell carcinoma (SCC) of the head and neck. Four months have passed since treatment was completed, and the patient remains recurrence-free both locally and in the neck (Figure 6).

## Discussion

Tongue-based tumors are generally SCC originating from the mucosa, with <0.5% of these malignancies arising from the salivary glands [4,5]. HCCC is a type of clear cell carcinoma (CCC) in which collagen fibrils in the tumor stroma are severely ventilated and form thick bundles. According to a recent review of 238 cases, the most common sites of occurrence include the palate (23.1%), tongue (21.0%), nasal cavity (10.5%), floor of mouth (6.3%), trachea (5.4%), parotid (4.2%), mandible (3.8%), lungs (3.3%), buccal mucosa (2.9%), lips (2.9%), maxilla (2.9%), posterior molar (1.7%), cheek (1.7%), and alveolar (1.3%) [6]. In addition, Hernandez et al. diagnosed clear cell carcinoma in approximately 2.5% of salivary gland malignancies over the past 20 years. HCCC is more common in women of relatively young age, with a median age of 57 years and an incidence of about 50-60% among women [7].

Tumors generally tend to develop slowly; therefore, symptoms are subjective, with local pain and discomfort being associated with tumor growth. In our case, the patient was aware of lymphadenopathy in the submandibular region and visited the clinic but only experienced mild discomfort due to the tumor at the base of the tongue, which was the primary lesion. Notably, many salivary gland neoplasms commonly or consistently have a component of clear cells. Therefore, HCCC in the head, neck, and oral cavity may be difficult to distinguish from other malignant tumors with clear cells. Consequently, a component of clear cells, such as epithelial-myoepithelial carcinoma, salivary myoepithelial carcinoma, and mucoepidermoid carcinoma (MEC), overlaps with these neoplasms and, until recently, has been distinguished by the absence of characteristic features of these tumors and their mononuclear cells [8].

The characteristics of these tumors and their monomorphous population of clear cells, such as CCC not otherwise specified (NOS), revealed a consistent EWSR1-Activating Transcription Factor 1 (ATF1) fusion in HCCC, and this molecular signature was not present in other clear cell mimics [7,9,10]. In previous reports, the positive rate of the EWSR1 fusion gene in HCCC was 82-92%, serving as a point of differentiation from other salivary gland tumors with pallid cells [11]. In pallid cell-rich myoepithelial and epithelioid myoepithelial carcinomas, the squamous cell markers p40 and p63 were positive, along with HCCC; however, HCCC did not usually show myoepithelial changes. MECs are the most common salivary gland malignancy and are a mixture of squamous, mucous, and intermediate cells [12]. Similar to that of HCCC, immunostaining of p40 and p63 was positive, while αSMA, calponin, and s-100 protein were negative. Recently, the CAMP-regulated transcriptional coactivator 1/3 (CRTC1/3)-mastermind-like transcriptional coactivator 2 (MAML2) fusion gene was found to be positive in 80% of MECs, and the EWSR1-ATF1 fusion gene was positive in over 90% of HCCC, which is a differentiating feature. Localization of the primary tumor also helps to differentiate HCCC from MEC, as most HCCC tumors tend to be located in the minor salivary glands of the oral cavity [6]. In contrast, MEC tumors tend to be located in the parotid gland and other major salivary glands [13]. When differentiating MEC from SCC, the squamous differentiation is prominent in SCC light-rich cells or CCC with poor vitalization, where p63 and p40 are positive, thus making it difficult to distinguish them [9].

Furthermore, some authors reported that p16 immunostaining was confirmed in all eight cases of CCC occurring in the mid-pharynx, of which two were strongly positive, and four of the eight cases were initially diagnosed as SCC or suspected SCC [14]. Human papillomavirus (HPV)-associated oropharyngeal carcinoma was ruled out because all cases were negative for high-risk HPV according to RNA-ISH staining, and the HCCC diagnosis was confirmed because all cases were positive for the EWSR1-ATF1 fusion gene [14]. Thus, it is more difficult to distinguish a primary oropharyngeal carcinoma that is p16-positive from SCC. Metastatic renal cell carcinoma is also a differential diagnosis; however, clinically, renal cell carcinoma rarely metastasizes to the head and neck region and does not have squamous cells. Thus, p63 and CK5/6 are negative. In this case, CK5/6, CK7, p40, and p63 were positive, while αSMA, calponin, and S-100 were negative, and the positive EWSR1-fluorescence in situ hybridization (FISH) staining of the preoperative biopsy tissue led to the HCCC diagnosis.

The mainstay of treatment for HCCC is extensive surgical resection. In previous review articles, surgical treatment was performed in 81.1% of 201 cases [6] and 144 of 153 cases (94.1%) [7]. Of these 144 patients, neck dissection was performed in 10.1% of the patients, and 26(18.1%), including 12 with positive preoperative LN; 25 (17.9%) patients received radiation or anticancer therapy, while 24 (16.7%) underwent radiotherapy alone, primarily for margin-positive and close by cases [6]. Chemotherapy was administered to two patients. The prognosis was good, with 5- and 10-year survival rates of 89% and 83%, respectively [7]. The 5-year local recurrence rate, cervical LN metastasis, and distant metastasis were 19%, 3%, and 2%, respectively. The final prognosis was death due to multiple distant metastases, including pulmonary and spinal metastases, in 3% [6] and three cases (2.0%) [7], with one case of local recurrence and lung metastasis. The remaining two cases were due to the rapid progression of the disease. In most cases, the disease could be controlled mainly by surgical treatment, and a good prognosis was maintained.

The following is a list of 12 cases of HCCC involving the base of the tongue, with detailed descriptions of the surgical approach [1-5,15-20] (Table 1). The base of the tongue, which is the anterior wall of the mid-pharynx, is one of the most common sites of HCCC after the soft palate [3,5]. However, surgical approaches to this region are difficult. In addition, the base of the tongue plays an important role in elevating the larynx for pharyngeal swallowing. Therefore, surgical resection of the base of the tongue should be performed with attention to postoperative swallowing function. The transmandibular approach, the traditional technique for treating tumors at the base of the tongue, is generally suitable for wide resection of large tumors, recurrent disease, advanced cancer not amenable to radiation chemotherapy, minor salivary gland tumors, and malignant lesions that are not radiosensitive [4,18]. It provides a wide field of view, allowing for a direct, clear view of the lesion, especially during the resection of malignant lesions, while confirming the margins [2,3,18]. It is also advantageous to reconstruct extensive resections, especially when combined with neck dissection [3].

**Table 1:** Clinical study on the surgical approach to HCCC of the base of the tongue ND: Neck dissection; AdCa: Adenocarcinoma; MEC: Mucoepidermoid carcinoma; DOD: Died of disease; NED: No evidence of disease; RT: Radiation therapy; CRT: Chemoradiation therapy; TOVS: Transoral video-assisted surgery; HCCC: Hyalizing clear cell carcinoma; POD: Postoperative day; N/A: Not available

Study	Age	Sex	Size	Diagnosis	Surgical approach	Reconstruction	ND	Tracheostomy	Discharge	Postoperative treatment	Prognosis
Milchgrub et al., 1994 [1]	51	F	3cm	HCCC	Total glossectomy laryngopharyngectomy	No	Ipsilateral	No	N/A	No	DOD
Balakrishnan et al., 2002 [4]	35	M	2cm	HCCC	Transmandibular approach	Local tongue flap	Ipsilateral	N/A	10 POD	No	NED 1Y
O’Regan et al., 2003 [16]	57	F	3.5cm	HCCC	Transmandibular approach	Forearm	Bilateral	N/A	1 Month	RT	DOD 10M lung and bone metastasis
Sicurella et al., 2004 [17]	69	N/A	3.5cm	HCCC	Transcervical approach	N/A	No	Yes	2 Weeks	No	NED 1Y
Suzuki et al., 2006 [2]	66	F	4.0cm	HCCC	Transmandibular approach	No	Ipsilateral	Yes	2 Weeks	No	NED 21M
Pujary et al., 2008 [15]	57	M	3cm	HCCC	Transcervical approach	No	Ipsilateral	No	3 Weeks	RT	NED 18M
Casani et al., 2011 [18]	75	F	2.2cm	HCCC	Transmandibular approach	No	Ipsilateral	Yes	8 POD	No	NED 18M
Watanabe et al., 2015 [19]	56	F	3.6cm	HCCC	Transoral approach	No	No	Yes	N/A	No	NED 15M
Chapman et al., 2018 [20]	68	F	3cm	HCCC	Transoral approach	No	Ipsilateral	No	N/A	No	NED 18M
Pillai et al., 2019 [5]	42	M	3cm	HCCC	Transoral approach	No	No	No	N/A	No	NED 1Y
AI Zadjali F et al., 2023 [3]	38	F	5.2cm	HCCC	Transmandibular approach	Forearm	Ipsilateral	Yes	12 POD	RT	NED 12M
Matsumoto et al., 2025	33	F	2.5cm	HCCC	TOVS	No	Bilateral	Yes	8 POD	CRT	NED 2M

In a previous report of 12 cases, the transmandibular approach was performed in five cases [2-4,16,18], and reconstruction was performed in three cases, including two radial forearm free flaps and one local tongue flap [3,4,16]. The tumor sizes ranged from 2-5.2 cm but were indicated in cases with large tumors, such as 4 cm and >5 cm in size. However, 2 cm tumor cases were observed [4,18]. In both cases, the preoperative diagnosis was HCCC, including clear cell adenocarcinoma, which was selected based on the recommendations for wide excision [4,18]. Other external incisional approaches, such as suprahyoid and lateral pharyngeal incisions, were performed in two cases [15,17]. Two cases were treated with the suprahyoid approach for relatively large tumors with tumor sizes of 3 and 3.5 cm. Suprahyoid pharyngectomy and lateral pharyngeal incisions do not require an incision on the floor of the mouth or an external incision on the lower lip. It is also advantageous in terms of cosmetics and speech compared to mandibulotomy. However, the disadvantage of this surgical technique is its limited field of view.

Recently, several cases of transoral resection have been reported, including small, superficial, well-defined lesions that are amenable to transoral resection using laser microsurgery or robotic surgery. Watanabe et al. performed transoral resection with tongue traction, and Chapman et al. performed transoral resection with transoral robotic surgery as an additional procedure after a definitive diagnosis was made using transoral excisional biopsy [19,20]. Pillai et al. used an oral approach similar to tonsillectomy with coblation for resection [5]. In this case, the authors initially considered extensive resection and reconstruction using a mandibulotomy approach because of the need for adequate margins similar to those for SCC of the oropharynx. In general, primary neoplasms of the base of the tongue are difficult to access through the oral cavity, particularly if they have a significant infiltrative effect. However, since the diagnosis of HCCC is preoperative and its characteristics include little tendency to invade, rare nuclear wrinkling and mitosis, and minimal histological changes, transoral excision is deemed feasible. The lesion was orally deployed using a retractor, placed under a specular view with the aid of an endoscope, and resected using an energy device and weak bending forceps. Pathology results showed rhabdomyosarcoma infiltration. However, the deep margins were negative.

For the treatment of HCCC, wide surgical margin excision of the primary tumor site is generally recommended, either with or without pre/postoperative radiotherapy [1,4,18,19]. In contrast, irradiation was performed in five (35.7%) of the 14 cases of HCCC of the base of the tongue, including this present case, which is higher than that reported in other regions. Three of the five cases, including this case, had multiple LN metastases, suggesting that irradiation was performed as a postoperative treatment indication for head and neck cancer in general. Furthermore, in this case, the primary tumor had a partially positive horizontal margin, the largest cervical LN metastasis was 6.5 cm in diameter, and extranodal invasion was observed. Therefore, chemoradiotherapy with weekly CDDP was judged to be indicated by standard post-treatment of SCC of the head and neck, and treatment was performed.

## Conclusions

HCCC is a rare, low-grade malignant tumor of the salivary glands, most commonly found at the base of the tongue. Surgery remains the primary treatment, and minimal surgery is one of the choices for indolent progressive tumors. In this case, a definitive diagnosis of HCCC was made preoperatively, and an oral surgical approach was adopted. Consequently, the patient experienced reduced hospital stays without postoperative functional impairment or complications. Minimally invasive surgical treatment should be considered, given the characteristics of HCCC, which differ from those of common squamous cell carcinomas of the oropharynx.

## References

[1] Milchgrub S, Gnepp DR, Vuitch F, Delgado R, Albores-Saavedra J (1994). Hyalinizing clear cell carcinoma of salivary gland. Am J Surg Pathol.

[2] Suzuki H, Katoh A, Udaka T, Shiomori T, Fujimura T, Fujimura K, Kitamura T (2006). Hyalinizing clear cell carcinoma arising from the base of the tongue. Acta Otolaryngol.

[3] Al Zadjali F, Alsaffar H, Odell M, Wasserman JK, Tohme A, Johnson-Obaseki S (2023). Base of the tongue hyalinizing clear cell carcinoma: Case report and literature review. SAGE Open Med Case Rep.

[4] Balakrishnan R, Nayak DR, Pillai S, Rao L (2002). Hyalinizing clear cell carcinoma of the base of the tongue. J Laryngol Otol.

[5] Pillai N, Balasundaram P, Isaac N (2019). Hyalinizing clear cell carcinoma: Base of tongue. Indian J Otolaryngol Head Neck Surg.

[6] Desai A, Faquin WC, Iafrate AJ, Rivera MN, Jaquinet A, Troulis MJ (2022). Clear cell carcinoma: A comprehensive literature review of 254 cases. Int J Oral Maxillofac Surg.

[7] Hernandez-Prera JC, Kwan R, Tripodi J, Chiosea S, Cordon-Cardo C, Najfeld V, Demicco EG (2017). Reappraising hyalinizing clear cell carcinoma: A population-based study with molecular confirmation. Head Neck.

[8] Hwang G, Goldenberg D, Warrick J, Slonimsky G (2020). A hyalinizing clear cell carcinoma of the base of tongue. Ear Nose Throat J.

[9] Cipriani NA, Kakkar A (2023). Top 10 clear cell head and neck lesions to contemplate. Head Neck Pathol.

[10] Weinreb I (2013). Hyalinizing clear cell carcinoma of salivary gland: A review and update. Head Neck Pathol.

[11] (2024). Publication of the WHO classification of tumours. https://www.iarc.who.int/news-events/publication-of-the-who-classification-of-tumours-5th-edition-volume-9-head-and-neck-tumours/.

[12] Peraza A, Gómez R, Beltran J, Amarista FJ (2020). Mucoepidermoid carcinoma. An update and review of the literature. J Stomatol Oral Maxillofac Surg.

[13] Hsieh MS, Wang H, Lee YH, Ko JY, Chang YL (2017). Reevaluation of MAML2 fusion-negative mucoepidermoid carcinoma: A subgroup being actually hyalinizing clear cell carcinoma of the salivary gland with EWSR1 translocation. Hum Pathol.

[14] Bishop JA, Rooper LM, Chiosea SI, Westra WH (2018). Clear cell carcinoma of salivary glands is frequently p16 positive: A pitfall in the interpretation of oropharyngeal biopsies. Am J Surg Pathol.

[15] Pujary K, Rangarajan S, Nayak DR, Balakrishnan R, Ramakrishnan V (2008). Hyalinizing clear cell carcinoma of the base of tongue. Int J Oral Maxillofac Surg.

[16] O'Regan E, Shandilya M, Gnepp DR, Timon C, Toner M (2004). Hyalinizing clear cell carcinoma of salivary gland: An aggressive variant. Oral Oncol.

[17] Sicurella F, Gregorio A, Stival P, Brenna A (2004). Clear cell carcinoma of minor salivary gland of the tongue. Acta Otorhinolaryngol Ital.

[18] Casani AP, Marchetti M, Seccia V, Fontanini G, Filice ME, Muscatello L (2011). Clear cell adenocarcinoma of the base of the tongue: A case report and review of the literature. Ear Nose Throat J.

[19] Watanabe K, Okumura Y, Hashimoto K, Suzuki T (2015). Clear cell carcinoma of the base of the tongue: Case report and literature review. Ann Otol Rhinol Laryngol.

[20] Chapman E, Skalova A, Ptakova N (2018). Molecular profiling of hyalinizing clear cell carcinomas revealed a subset of tumors harboring a novel EWSR1-CREM fusion: Report of 3 cases. Am J Surg Pathol.

